# Social–Emotional Competence Growth Profiles in Upper Elementary School Years and Pathways to Mental Health Outcomes in Middle School †

**DOI:** 10.3390/ijerph22111744

**Published:** 2025-11-18

**Authors:** Juyeon Lee, Chenxiao Wang

**Affiliations:** 1 Department of Social Work and Social Administration, The University of Hong Kong, Hong Kong SAR, China; 2 Department of Human Development and Family Studies, University of Wisconsin-Madison, Madison, WI 53706, USA; chenxiao.wang@wisc.edu

**Keywords:** social-emotional competence (SEC), mental health, adolescence, middle school transition, latent profile growth modeling, mediation analysis, Korean Children and Youth Panel Survey 2018 (KCYPS 2018)

## Abstract

Social–emotional competence (SEC) is an essential factor for healthy youth development. However, few studies have examined patterns of SEC growth trajectories among non-Western youth, and whether and how their SEC growth patterns during elementary school years predict later mental health. Using five-year panel data on a nationally representative sample of South Korean youth (*N* = 2607; 49.6% girl, M_age_ = 10, SD_age_ = 0.1 at baseline), we first identified three latent profiles of SEC growth trajectories throughout upper elementary years (Grades 4 to 6), distinguished by initial and continued mean-level differences in both self-management and group collaboration. Informed by self-determination theory, we found that these SEC growth profiles significantly predicted depression and life satisfaction in middle school (Grade 8), mediated by peer relatedness and academic competence during the middle school transition (Grade 7). This study discusses implications for future research and practice to promote young adolescents’ social–emotional development and mental health.

## 1. Introduction

Adolescent mental health problems are a global public health concern. A meta-analysis of the global prevalence of depressive symptoms suggests that 34% of teenagers were at risk of developing clinical depression from 2001 to 2020, with an increasing trend from 24% to 38% across the two decades [1]. The Global Burden of Disease Study found that around 14% of teenagers, worldwide, experienced a mental disorder such as depression and anxiety in 2019 [2]. During the COVID-19 pandemic, the estimated prevalence rates increased up to 25.2% for clinically elevated depression and 20.5% for clinically elevated anxiety, according to another meta-analysis study [3]. All these findings warrant the need to accumulate scientific evidence that can inform societal efforts to prevent mental health problems and promote the well-being of adolescents.

The transition from elementary to middle school is a particularly critical period for youth mental health, as most young adolescents face heightened risks due to the multidimensional changes they undergo in this period [4,5]. Given that about half of all lifetime mental disorder cases start by age 14 [6], maintaining good mental health during the middle school years can serve as a foundation for optimal development and well-being throughout the rest of adolescence and beyond [7]. From a preventionist standpoint, it is crucial to provide early support to cultivate protective and promotive factors of mental health before the middle school transition period.

Decades of research have demonstrated that social–emotional competence (SEC) is an important protective and promotive factor of healthy youth development, which is also malleable throughout the school years, particularly during the elementary school years [8,9,10]. A body of experimental and meta-analytic evidence has shown that school-based social and emotional learning (SEL) interventions aimed at promoting student SEC have positive effects on youth mental, behavioral, and academic outcomes [11,12]. Based on this, various local and international entities have advocated for a wide dissemination of school-based social–emotional learning (SEL) interventions to promote youth mental health across the world [13].

### 1.1. Development of Social–Emotional Competence (SEC) in Upper Elementary School Years

SEC is a multidimensional construct involving diverse skills and mindsets that enable an individual to engage in positive and effective interactions with oneself and others in certain cultural contexts [14,15]. SEC often refers to a set of emotional, cognitive, and behavioral abilities that contribute to both intrapersonal and interpersonal well-being and functioning [10,16]. Among the various aspects of SEC, self-management (e.g., emotional and behavioral regulation) and positive social behavior (e.g., prosocial and collaborative behavior) have been the two most frequently studied components of SEC, commonly emphasized across different conceptual frameworks of SEC such as the CASEL framework [17] and the Big Five model [18]. Self-management is often indicated by the abilities to control one’s emotions, thoughts, and behavior in an effective way, while positive social behavior is often indicated by the abilities to interact with other people in a prosocial and collaborative way [17,18]. SEC is not a fixed trait; it develops over time within social environments. The emergence and development of social and emotional abilities in early childhood have long been an important topic in developmental science. The literature suggests that, overall, toddlers and preschoolers tend to show more socially and emotionally competent behaviors as they grow older [16,19,20,21,22], although the observed extent of the changes has varied across studies [23,24,25].

The literature on SEC development during the school-age years has been relatively less in volume and more inconclusive than studies of early childhood [26]. The available studies tend to report generally stable or slightly declining trajectories of socially competent behavior throughout the elementary school years under non-intervention conditions [27,28]. These studies have also explored within-sample heterogeneity in the observed growth patterns, typically identifying two or three distinct trajectories characterized by initial and continued mean-level differences across multiple school years [27]. Some recent studies, however, have found an overall increasing trajectory of SEC-related skills during the upper elementary school years and beyond [29,30]. These studies have also suggested that specific sub-constructs of SEC may show distinct growth trajectories [29] and that there may exist more heterogeneous subgroups of students showing different patterns of increases or decreases in SEC over time [30]. Building on the preceding literature, this study hypothesizes that different subgroups of school-age children may display heterogeneous growth patterns in SEC. This calls for the use of a person-centered approach (e.g., examining latent growth profiles) rather than a variable-centered approach (e.g., comparing average levels across time) to identify these subgroups.

One of the critical but under-recognized gaps in the literature is that the current knowledge base on SEC developmental trajectories predominantly relies on studies conducted in Western countries. Despite methodological differences such as sample characteristics or measures, all of the above-reviewed studies were conducted within North American or European countries. It remains largely unanswered how SEC develops during the school-age years in other parts of the world. For example, according to a recent systematic review of 45 empirical studies on social–emotional development of Asian children and adolescents, most of them adopted a cross-sectional design or a short-term pretest-posttest design, while no study that examined social–emotional developmental trajectories across multiple years was identified [31].

Given the broad literature suggesting that Eastern culture generally emphasizes self-control and social harmony more than Western culture [32,33], one might expect to find somewhat different patterns of SEC growth among Asian youth from what has been typically observed in previous research. However, there is no sufficient evidence to inform our knowledge base or research hypothesis on typical SEC developmental trajectories among Asian youth. The first aim of this study was to address this gap by exploring patterns of normative growth trajectories of SEC during the upper elementary school years using a nationally representative sample of Korean youth. In doing so, the two key components of SEC, self-management and group collaboration, were considered separately but simultaneously to get a more comprehensive picture of SEC development in upper elementary school years. This would set the stage for further examinations of whether and how these SEC growth trajectories predict later mental health outcomes in adolescence.

### 1.2. Pathways from Earlier SEC Development to Later Mental Health

Previous literature has suggested that earlier SEC predicts later mental health outcomes. For example, emotional competence or prosocial skills assessed at around age 5–6 was found to predict mental health outcomes (e.g., internalization problems, life satisfaction) later in the school-age years [34,35] and even in young adulthood [36]. One study focused on adolescence found that temperance (representing emotional and behavioral regulation) and interpersonal strengths (representing communion, collectivism, and convivial relationships) assessed at Grade 7 predicted subjective well-being and middle school adjustment at Grade 8 [37].

Less research has been conducted to explain the mediational mechanisms underlying the relations between earlier SEC and later mental health outcomes. Available evidence suggests that the effects of SEC on mental health during the middle school transition period could be mediated by variables related to academic and social factors. For example, a short-term longitudinal study found that self-regulatory beliefs predicted stress and depression among American students experiencing the middle school transition, mediated through academic disengagement [38]. Recently, more broadly defined SEC was reported to be associated with mental health outcomes, such as depression and emotional distress, mediated through social support among Korean upper elementary school students [39] or through academic efficacy beliefs among Norwegian middle school students [40]. However, the mediational claims of these studies were limited due to cross-sectional designs.

According to self-determination theory (SDT) [41], autonomy, competence, and relatedness are the three basic psychological needs for human motivation and well-being. During the middle school transition, fulfilling the needs of autonomy, competence, and relatedness within the academic and peer-related domains may become particularly important, as young adolescents going through this period typically experience increased pressure and desires to show good academic performance and build positive peer relationships [4,5]. For example, adolescents who perceive themselves to have freedom and control over academic learning (i.e., whose needs for academic autonomy are met), those who perceive themselves to be capable of achieving their academic goals (i.e., whose needs for academic competence are met), and those who perceive themselves to have positive relationships with their peers (i.e., whose needs for peer relatedness are met) are more likely to show better mental health outcomes than those not. Previous studies based on SDT have empirically supported this idea with secondary school and university students, suggesting that their perceived academic autonomy, academic competence, and peer relatedness were, together, related to various positive outcomes, including subjective well-being [42,43]. The broader literature has also suggested that mental health during adolescence can be explained by academic autonomy [44,45], academic competence [46,47], and peer relationships [48,49,50].

In the meantime, SEC, such as self-management and positive social behavior, seems to be an important factor that enables adolescents to fulfill these three basic psychological needs. Regarding academic autonomy, student SEC has been correlated with autonomous motivation [51] and academic engagement [52] among secondary and university students. Regarding academic competence, longitudinal research has suggested that earlier SEC predicted later academic achievement during the elementary school transition [53] as well as the middle school transition [49]. Regarding peer relatedness, the effects of earlier SEC on later peer relationships during childhood and adolescence have also been supported by longitudinal research [35,54]. Although most studies that examined the roles of SEC in the fulfillment of these three basic psychological needs were conducted in the Western societies, there exist some studies that reported the effects of SEC on academic achievement and peer relationships among East Asian students [55,56].

Based on the previous literature suggesting the relations among SEC, mental health, and the three psychological needs according to SDT, it seems reasonable to hypothesize that autonomy, competence, and relatedness, especially within the academic and peer-related domains, would mediate the relation between earlier SEC development and later mental health outcomes in adolescence. As noted in Vestad et al.’s research [40], SEC may help young adolescents meet their psychological needs, such as academic autonomy, academic competence, and peer relatedness, which in turn can prevent later mental health problems and promote their mental well-being.

Despite the conceptual plausibility, there has been a lack of research that examines a path model linking earlier SEC to later mental health during adolescence through academic autonomy, academic competence, and peer relatedness. The second aim of this study was to examine whether and how SEC growth profiles in the upper elementary school years predicted mental health outcomes in adolescence among Korean youth. Specifically, this study focuses on the indirect pathways through academic autonomy, academic competence, and peer relatedness during the middle school transition period.

### 1.3. Research Objectives

**Figure 1:**
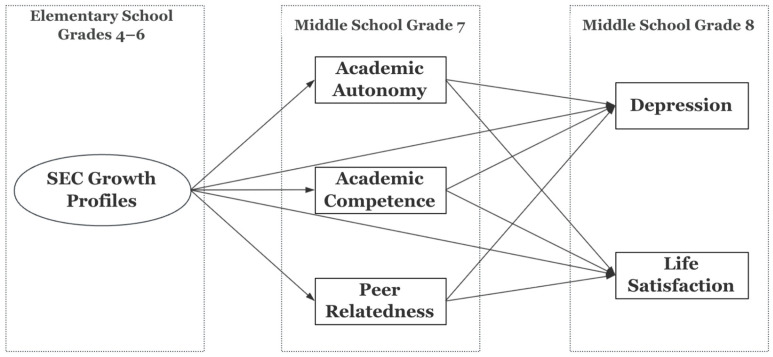
The Conceptual Model of the Relations. The model depicts the relations among SEC growth trajectory profiles in upper elementary school years, three basic psychological needs during the middle school transition, and adolescent mental health outcomes. The effects of covariates and the correlations among three mediators and between two outcome variables are not presented in this figure for improved legibility.

The main objective of this study is twofold: (1) to identify latent profiles of SEC growth trajectories during the upper elementary school years among Korean youth, and (2) to examine the effects of these SEC growth profiles on later mental health outcomes in middle school, focusing on the mediational roles of academic autonomy, academic competence, and peer relatedness during the middle school transition period. Figure 1 presents the conceptual model of this study. Various covariates and potential confounders were controlled for in the actual analysis, including baseline measures of mediators and outcome variables as well as student sociodemographic characteristics and environmental factors such as parenting and teacher relationships. More detailed information is presented in the Section 2.

## 2. Materials and Methods

### 2.1. Data and Sample

The dataset used in the study comes from the Korean Children and Youth Panel Survey (KCYPS) 2018, a nationally representative longitudinal survey in South Korea that includes multidimensional information on children’s and adolescents’ development and well-being throughout their school years. Using a multi-stage stratified cluster sampling method, KCYPS 2018 began the first wave of data collection in 2018 with two age cohorts: a younger cohort comprising Grade 4 elementary school students and an older cohort comprising Grade 7 middle school students at baseline. The study has tracked these same participants annually over time.

In line with our focus, the current study analyzed the five waves (2018–2022) of the KCYPS 2018 data collected from the younger cohort, covering their three upper elementary school years (Grades 4 to 6) and the first two years in middle school (Grades 7 and 8). The KCYPS 2018 younger cohort comprised 2607 students in Wave 1 (2018, Grade 4) and was followed annually, with 2437 students retained in Wave 2 (2019, Grade 5), 2411 students in Wave 3 (2020; Grade 6), 2275 students in Wave 4 (2021; Grade 7), and 2311 students in Wave 5 (2022; Grade 8). The five-year sample attrition rate was 11.35%. The results from Little’s Missing Completely at Random (MCAR) tests with all the major variables of this study and the key demographic variables (i.e., age and gender) of the sample suggested that there was no serious violation to the MCAR assumption (χ^2^ = 149.00, *df* = 127, *p* = 0.09). We also applied *t*-tests to compare the baseline measures of the main study variables, finding non-significant differences (all *p* > 0.10) between the sample retained after five years (*N* = 2311) and the sample lost to attrition (*n* = 296). Based on these results, the full baseline sample (*N* = 2607) was used for further analyses, applying the Full Information Maximum Likelihood (FIML) approach to handle missing data. Supplementary Table S1 presents the baseline sociodemographic characteristics of the sample.

### 2.2. Measurements

#### 2.2.1. Mental Health

The current study focuses on both negative and positive outcomes of mental health: depression and life satisfaction. In the fifth wave of the data (in 2022, when participants were in Grade 8 or the second year in middle school), depression was measured by ten items (e.g., “I feel unhappy, sad, and depressed,” “I worry a lot,” “I have no interest or enthusiasm for anything,” “I have thoughts of wanting to die”; α = 0.89), which have been validated and used to assess Korean adolescents’ depressive symptoms [57,58]. Life satisfaction was measured by five items (e.g., “I am satisfied with my life,” “If I could live my life again, I would change almost nothing”; α = 0.81) using Diener et al.’s Satisfaction with Life Scale [59], which has been assessed as a valid and reliable measure in Korean population [60]. All the items were measured on a 4-point Likert scale, ranging from 1 (not at all) to 4 (very much). In the current study, mean composite variables were calculated for depression and life satisfaction, with higher values indicating greater levels of depression and life satisfaction, respectively. The descriptive statistics of these two mental health outcome variables are presented in Supplementary Table S2.

#### 2.2.2. Social–Emotional Competence

The current study focuses on two components of SEC: self-management and group collaboration. First, this study defines self-management as the ability to regulate one’s attention, emotion, and behavior in a way that helps successful task completion and positive social interaction. In the first three waves of KCYPS 2018 (in 2018 to 2020, when participants were Grades 4–6 in elementary school), self-management was assessed using three indicators: (1) attention control (measured by seven reverse-coded items originally assessing attention deficit; e.g., “I don’t want to do a task that requires a long time of concentration,” “I make mistakes or trouble because I don’t pay attention”) [60], (2) aggression control (measured by six reverse-coded items originally assessing aggression; e.g., “There are days when I feel angry all day,” “If someone prevents me from doing what I want, I argue or fight”) [61], and (3) grit (measured by eight items indicating passion or diligence, interest or perseverance, and concentration of effort with four reverse-coded items; e.g., “Once I start something, I always finish it,” “I don’t get too frustrated when facing difficulties while trying to solve a problem, and “I tend to bounce back from setbacks faster than others”) [62]. All the items were measured on a 4-point Likert scale, ranging from 1 (not at all) to 4 (very much). The composite mean scores of these three indicators of self-management had moderate-to-high correlations with each other at each wave (r = [0.42, 0.67]; α = 0.89).

Second, this study defines group collaboration as the ability to collaborate with others in a way that contributes to a shared goal and problem-solving in group activities. In the first three waves of KCYPS 2018, group collaboration was assessed using three indicators [63]: (1) followership (measured by three items; e.g., “I do my best in my role when participating in group activities”), (2) group facilitation (measured by three items; e.g., “When participating in group activities, I create an atmosphere where my friends feel comfortable expressing their opinions freely,” “When my friends are struggling during group activities, I willingly offer my help”), and (3) group problem-solving (measured by five items; e.g., “I work with friends to solve important issues or problems that arise during group activities,” “When problems arise in group activities, I try to find solutions rather than pointing out who is to blame”). All the items were measured on a 4-point Likert scale, ranging from 1 (not at all) to 4 (very much). The composite mean scores of these three indicators of group collaboration had high correlations with each other at each wave (r = [0.69, 0.90]; α = [0.92, 0.93]).

Exploratory factor analysis with the six composite variables described above supported the two-dimensional structure of SEC across all three waves, clustering attention control, aggression control, and grit into one factor, while clustering followership, group facilitation, and group problem-solving into another factor. Confirmatory factor analysis also supported the validity of this two-dimensional SEC measurement model using six composite variables from all three waves, suggesting an acceptable model fit (χ^2^/*df* = 14.04, *p* < 0.001; CFI = 0.93; TLI = 0.91; SRMR = 0.05; RMSEA = 0.075). In addition, the results of the longitudinal measurement invariance test suggest strong measurement invariance (i.e., configural, metric, and scalar invariance) across three waves, based on the negligible model fit differences (i.e., ΔCFI < 0.01, ΔTLI < 0.01, ΔRMSEA < 0.01, and ΔSRMR < 0.01).

Given the complexity of our research models, a set of two composite variables were calculated for self-management and group collaboration at each wave by averaging the scores of relevant items in a way that higher values indicate greater self-management and group collaboration skills, respectively. The descriptive statistics of self-management and group collaboration variables across three waves are presented in Supplementary Table S2. These two composite variables, repeatedly measured from Waves 1 to 3, were used in the following analysis to identify patterns of SEC development among students during their upper three elementary school years.

#### 2.2.3. Academic Autonomy, Academic Competence, and Peer Relatedness

The current study used relevant indicators of academic autonomy, academic competence, and peer relatedness, measured in the fourth wave of KCYPS 2018, which was collected in 2021 when the participants were in Grade 7 or the first grade in middle school.

Academic autonomy was measured by four reverse-coded items originally assessing the lack of self-directed academic engagement (e.g., “I do not study on my own initiative,” “I have never tried to make a study plan on my own”; α = 0.80) on a 4-point Likert scale (1 = “not at all” to 4 = “very much”) [64]. Academic competence was measured by two items asking one’s subjective evaluation of and satisfaction with overall academic performance in the first semester of middle school (α = 0.78). Both items were measured on a 5-point Likert scale (1 = “very poor” or “very dissatisfied” to 5 = “very good” or “very satisfied”) [58]. Peer relatedness was measured by eight items measuring positive peer relationships (e.g., “I have a good relationship with my friends,” “I can share my secrets with my friends,” “My friends care about me”; α = 0.87) on a 4-point Likert scale (1 = “not at all” to 4 = “very much”) [65]. In the current study, a mean composite variable was calculated for each of these variables, with higher values indicating greater levels of academic autonomy, academic competence, and peer relatedness, respectively. The descriptive statistics of these three variables are presented in Supplementary Table S2.

#### 2.2.4. Covariates

To statistically control for potential confounders, several covariates were included in the analysis of the mediational model. First, all the baseline measures of the mediators and outcome variables were included as a predictor of the later corresponding variable. The basic descriptive statistics of these covariates measured at Wave 1 are as follows: depression (M = 1.53, SD = 0.53, α = 0.89), life satisfaction (M = 3.10, SD = 0.57, α = 0.81), academic competence (M = 3.99, SD = 0.75, α = 0.80), and peer relatedness (M = 2.98, SD = 0.52, α = 0.84). Academic autonomy was not measured during the elementary school years, thus could not be included as a covariate. In addition, student individual and environmental characteristics that could also be simultaneously associated with our main study variables were included as covariates, including student gender (girl or boy), self-rated physical health (4-point Likert scale), number of siblings (including oneself), parent-reported family income level (grouped into 12 levels), and school location (urban vs. rural), as well as student-reported positive parenting and teacher relationships. Positive parenting was measured as a mean composite variable of 12 items assessing parental warmth, autonomy support, and structure provision (M = 3.46, SD = 0.45, α = 0.90) [66], while teacher relationships was measured as a mean composite variable of 14 items assessing teachers’ acceptance, trustworthiness, accessibility, and sensitivity (M = 3.00, SD = 0.49, α = 0.91) [58], both of which were measured on a 4-point Likert scale. Except for the number of siblings collected in Wave 2, all of the other variables were all baseline measures.

### 2.3. Analysis Methods

#### 2.3.1. Latent Profile Growth Modeling (LPGM)

To address the first research objective, latent profile growth modeling (LPGM) was conducted to identify different subgroups underlying SEC growth trajectories. This person-centered approach is aligned with our study’s focus on within-sample heterogeneity in SEC developmental trajectories, considering both the intercept (i.e., baseline level) and slope (i.e., growth rate) across multiple time points to identify subgroups with disparate growth profiles [67]. Also, the results of LPGM, or the membership in the estimated latent profiles that consider both intercept and slope of growth trajectories, could be used as a categorical predictor for later outcomes in the following analysis. Besides, compared to other growth modeling, LPGM is recommended for longitudinal studies with a large sample size, which is applicable to the data used in the study [67,68].

In a stepwise approach, we started with an unconditional model assuming one latent profile followed by models assuming an increasing number of profiles to test the relative fit of these competing models. To determine the optimal number of profiles, model fit indices were evaluated, along with the interpretability of each model. In line with suggestions from previous research, a set of fit indices were taken into consideration, including the Akaike information criterion (AIC), Bayesian information criteria (BIC) [69], adjusted Bayesian information criteria (aBIC) [70], classification accuracy (i.e., entropy), the Lo–Mendell–Rubin likelihood ratio test (LMRT) *p*-values [71], and bootstrap likelihood ratio test (BLRT) *p*-values [72]. A better model fit was indicated by lower values on AIC, BIC, and aBIC and higher values on entropy (ranging from 0 to 1 with higher values indicating higher classification accuracy). In addition, a *p*-value less than 0.05 for LMRT and BLRT indicates a significant improvement of the model fit as a result of having one more profile. Finally, the size of each profile was also taken into consideration to enhance the interpretability and replicability of the findings: only models with all the profile sizes of at least 5% of the total sample were considered. In each model estimation, starting points were randomly chosen. The mean intercepts and slopes of the two SEC variables were constrained to have no variance in each profile. LPGM was conducted using Mplus (version 8.3) with the Maximum Likelihood with Robust Standard Errors (MLR) estimator to obtain robust parameter estimates and handle missing data based on the FIML method.

#### 2.3.2. Mediation Analysis

In addition to exploring the heterogeneous profiles of SEC growth trajectories in upper elementary schools, the LPGM approach allows the use of these profiles as variables embedded in a more complex model. To address the second research objective, a structural equation model was conducted to examine the proposed mediation model. The adequacy of the model fit was first evaluated using several fit indices, including the Chi-Square statistic (χ^2^), Comparative Fit Index (CFI), Tucker–Lewis Index (TLI), the root-mean-square error of approximation (RMSEA), and the standardized root-mean-square residual (SRMR) according to the conventional guidelines for assessing a good fit (e.g., CFI and TLI above 0.90, RMSEA and SRMR < 0.05) [73]. Then, the direct, indirect, and total effects of the major paths were estimated and tested to verify whether the indirect effects via the mediators were significant. Based on the suggestion of [74], the significance of indirect effects was tested using Bootstrap with 1000 replicates to calculate the 95% confidence interval (CI). The mediating effects were interpreted to be significant at the alpha level of 0.05 when these confidence intervals did not contain zero. Mediation analysis was conducted with the lavaan package (version 0.6–16) [75] in Rstudio (version 2023.06.1+524), using FIML-based MLR estimator.

## 3. Results

### 3.1. Latent Profiles of SEC Growth Trajectories

Before conducting the main analyses, we first examined the bivariate correlations among the key variables in the current study. As shown in Supplementary Table S3, the correlations between the repeated measures of the same SEC variables across three waves ranged from 0.34 to 0.44 (*p* < 0.001). Based on the LPGM results, a three-profile model was finally selected to be a best-fit model of SEC growth profiles. Supplementary Table S4 shows the model fit and group size of each profile for one- to four-profile models. Although the four-profile model had the lowest values of AIC, BIC, and aBIC and the highest value on entropy, the smallest profile size was below 5% of the sample size (*n* = 70), leading to difficulty in interpretability. In contrast, the three-profile model had the second-lowest scores on AIC, BIC, and aBIC. Additionally, although the two-profile model had a higher value on entropy than the three-profile model, the significant LMR and BLRT of the three-profile model indicated better classification accuracy for the three-profile model. Furthermore, the three-profile model had the most meaningful classifications with self-management and group collaboration variables. Therefore, the three-profile model was selected as the final solution in the study.

In the three-profile model, the three subgroups of students were identified and named as the Profile with medium SEC, the Profile with low SEC, and the Profile with high SEC, respectively. Although an adequate group size was achieved for each profile, the distribution was not balanced, with 55.47% of the total sample classified into the Profile with medium SEC (*n* = 1446), whereas 27.08% of students were classified into the Profile with low SEC (*n* = 682) and 17.45% into the Profile with high SEC (*n* = 479).

**Figure 2:**
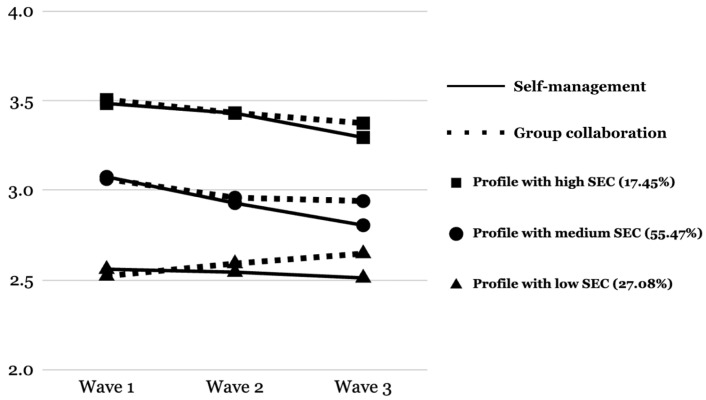
Changes in Mean SEC Scores Over Time Across Three Latent Subgroups (*N* = 2607).

As shown in Figure 2, these three subgroups showed significant level differences in both self-management and group collaboration consistently throughout their upper elementary school years. In other words, students in the Profile with high SEC showed consistently higher levels of self-management and group collaboration than those in the Profile with medium SEC, while students in the Profile with low SEC showed consistently lower levels of self-management and group collaboration than those in the Profile with medium SEC.

Furthermore, different growth trends in both self-management and group collaboration were observed across these subgroups. Students in the Profiles with medium and high SEC had a significant decrease in both self-management and group collaboration throughout the three elementary school years, with self-management showing more decline than group collaboration. The results from the *t*-tests comparing estimates of growth rate suggested that the rate of decrease in self-management was larger for the Profile with medium SEC than for the Profile with high SEC, while the rates of decrease in group collaboration were not statistically different between the two subgroups. Meanwhile, students in the Profile with low SEC showed somewhat different patterns of SEC growth. They also showed a significant decrease in self-management, but the rate of change was smaller than the other two subgroups, leading to a more stable trajectory. Unlike the other two subgroups, students in the Profile with low SEC had a significant increase in group collaboration.

The three SEC growth profile subgroups showed significant differences in sociodemographic and baseline characteristics, as presented in Supplementary Table S5. There were more boys (55%) in the Profile with low SEC and girls (57%) in the Profile with high SEC. Physical health, family income level, positive parenting, teacher relationships, and the baseline measures of mediators and outcome variables were also found to be associated with the level of SEC. These differences were statistically controlled for when conducting mediation analysis.

### 3.2. Mediation Model

Based on the LPGM results, two dummy variables for the Profiles with low and high SEC were created, setting the Profile with medium SEC as a reference group. These variables indicating different patterns of early SEC development trajectories were then used as main predictors of mental health outcomes in adolescence. The proposed mediation model showed a good fit to the data: χ^2^ = 34.04, *df* = 16, *p* = 0.005; RMSEA = 0.02; CFI = 0.99; TLI = 0.94; SRMR = 0.01.

**Figure 3:**
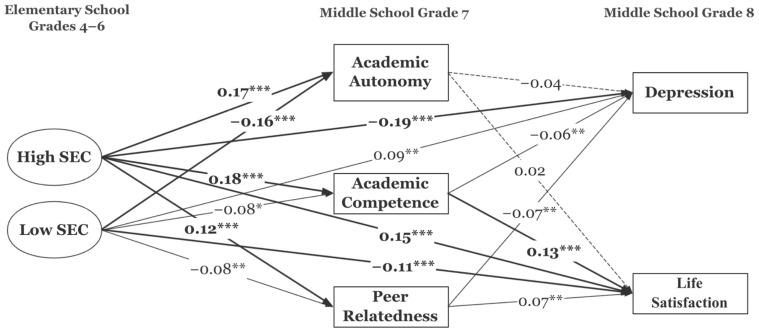
Standardized Regression Coefficients of the Mediation Model. * *p* < 0.05, ** *p* < 0.01, *** *p* < 0.001 (two-tailed); The correlations and the effects of covariates are not presented in this figure for improved legibility.

All of the estimated regression coefficients and the associated *p*-values and confidence intervals are presented in Supplementary Table S6. Figure 3 presents the standardized coefficients of the direct paths among the main variables after adjusting for covariates. Compared to the Profile with medium SEC, students who had consistently higher SEC growth trajectories in their upper elementary school years showed higher levels of academic autonomy (β = 0.11, *p* < 0.001), academic competence (β = 0.10, *p* < 0.001), and peer relatedness (β = 0.09, *p* < 0.001) in the first year of middle school. In contrast, students who had consistently lower SEC growth trajectories in their upper elementary school years showed lower levels of academic autonomy (β = −0.12, *p* < 0.001), academic competence (β = −0.05, *p* < 0.05), and peer relatedness (β = −0.07, *p* < 0.01) in the first year of middle school. In the first two middle school years, earlier academic autonomy did not predict life satisfaction and marginally predicted depression (β = −0.05, *p* = 0.05, 95% CI [−0.089, −0.002]). Earlier academic competence predicted both depression (β = −0.08, *p* < 0.01) and life satisfaction (β = 0.18, *p* < 0.001). Earlier peer relatedness predicted both depression (β = −0.07, *p* < 0.01) and life satisfaction (β = 0.06, *p* < 0.01).

Bootstrapping was conducted to test the significance of indirect effects as well as total effects. Supplementary Table S6 also presents the estimates and bootstrapped confidence intervals for indirect and total effects of the main relations. First, those who had consistently higher SEC growth trajectories in their upper elementary school years had significantly lower levels of depression in middle school (total effects β = −0.14, 95% CI [−0.249, −0.130]). The effects of high SEC on depression were both direct (β = −0.12, *p* < 0.001) and indirect through academic autonomy (although marginal; β = −0.01, 95% CI [−0.016, 0.000]), academic competence (β = −0.01, 95% CI [−0.020, −0.003]), and peer relatedness (β = −0.01, 95% CI [−0.016, −0.002]).

Also, those who had consistently higher SEC growth trajectories in their upper elementary school years had significantly higher levels of life satisfaction in middle school (total effects β = 0.11, 95% CI [0.083, 0.217]). The effects of high SEC on life satisfaction were both direct (β = 0.08, *p* < 0.001) and indirect through academic autonomy (although marginal; β = −0.01, 95% CI [0.000, 0.016], academic competence (although marginal; β = −0.01, 95% CI [0.000, 0.011], and peer relatedness (β = 0.01, 95% CI [0.001, 0.012]).

In the meantime, those who had consistently lower SEC growth trajectories in their upper elementary school years had significantly higher levels of depression (total effects β = 0.07, 95% CI [0.027, 0.144]). The effects of low SEC on depression were both direct (β = 0.55, *p* = 0.033) and indirect through academic competence (β = 0.01, 95% CI [−0.023, −0.011]) and peer relatedness (β = 0.004, 95% CI [−0.013, −0.001].

Further, those who had consistently lower SEC growth trajectories in their upper elementary school years had significantly lower levels of life satisfaction (total effects β = −0.09, 95% CI [−0.170, −0.055]). The effects of low SEC on life satisfaction were both direct (β = −0.07, *p* < 0.01) and indirect through academic competence (β = 0.02, 95% CI [0.011, 0.038]) and peer relatedness (β = 0.01, 95% CI [−0.001, −0.016]).

## 4. Discussion

The objective of this study was to explore the patterns of normative SEC development in upper elementary school years and examine the indirect pathways linking these SEC growth patterns to later mental health outcomes in middle school. Using nationally representative panel data collected from Korean youth from Grades 4 to 8, this study aimed to (1) identify latent profiles of SEC growth trajectories during the upper elementary school years, and (2) examine the effects of these SEC growth profiles on later mental health outcomes, focusing on the mediational roles of academic autonomy, academic competence, and peer relatedness during the middle school transition.

### 4.1. SEC Development in Upper Elementary School Years

Regarding the first aim of this study, the LPGM results identified three distinct SEC trajectory profiles (i.e., with low, medium, and high SEC), primarily distinguished by initial and sustained level differences in self-management and group collaboration. This finding is aligned with the previous studies on Western youth that also found heterogeneous SEC growth patterns characterized by overall level differences across multiple school years [27]. Overall, two SEC indicators, particularly self-management, declined from Grades 4 to 6, although students in the low SEC profile showed a comparatively smaller decline in self-management and a slight increase in group collaboration. Despite this, they still demonstrated the lowest SEC levels by Grade 6. While this result is largely consistent with the previous studies that reported overall declining trajectories of SEC during the school-age years [27,28,76], this study provides more nuanced findings by using two indicators of SEC separately but simultaneously and also considering within-sample heterogeneity.

While all these findings contribute to the limited literature on SEC growth trajectories during the elementary school years, from a practical perspective, this study raises a question about what we can and should do to reverse the declining trajectories of SEC, overall, as well as to close the existing disparities in these trajectories among Korean youth. Previous research conducted within the U.S. school context has suggested that elementary school students receiving universal SEL interventions show overall increases in SEC [77,78], and while initial gaps may not be significantly reduced, SEL can help prevent these disparities from widening over time [78,79]. Recently, the Ministry of Education of South Korea has initiated efforts to integrate universal SEL across elementary to high school systems [80]. In this context, the findings of the current study call for more research to examine whether and how school-based SEL interventions can not only promote Korean students’ SEC development in general but also yield more equitable outcomes.

### 4.2. Mediational Pathways from Early SEC to Adolescent Mental Health

Regarding the second aim of this study, the mediation model demonstrated a good fit and supported prior longitudinal findings [34,35,36,37]: youth with consistently higher SEC in their upper elementary school years had lower depression and greater life satisfaction in middle school. Academic competence and peer relatedness during the transition year mediated these associations, such that higher self-management and group collaboration predicted stronger academic and peer adjustments, which in turn related to better mental health outcomes. Interestingly, academic autonomy was found to be an additional, albeit marginal, mediator that distinguished only between the medium and high SEC profiles, suggesting that academic autonomy may further enhance well-being among youth with already elevated levels of SEC.

Overall, these findings are generally consistent with the previously reviewed studies that examined the direct relations between major variables of interest. Particularly, this study supports the existing literature suggesting the importance of SEC in academic achievement and peer relationships among East Asian students [55,56] and the importance of academic achievement and peer relationships in Korean adolescents’ mental health [39,50,81]. This study, however, made unique contributions to the literature by empirically examining a comprehensive path model using longitudinal data. As suggested by the literature on SDT [40], SEC growth profiles in the upper elementary school years were related to mental health in adolescence, partially by satisfying the three major psychological needs in the middle school transition: academic autonomy, academic competence, and peer relatedness [4,5,82].

To translate this empirical research evidence into practice and policy, the findings of our study provide important implications regarding how to support Korean students during their transition to middle school—a period often characterized by heightened academic and social stressors that pose risks to student mental health within a highly competitive educational context [83,84]. Notably, the recent policy-level introduction and endorsement of school-based SEL in Korea was, in part, a response to the prevalent mental health problems among Korean children and adolescents [80]. The findings of the current study offer empirical support for this policy direction and valuable insights into SEL practice strategies, particularly for young adolescents. Specifically, cultivating self-management and group collaboration skills among upper elementary school students may better prepare them to meet academic demands and navigate peer relationships during the middle school transition, thereby leading to a smoother adjustment and more positive mental health outcomes. Moreover, the comparisons across subgroups with distinct SEC growth profiles suggest that the benefits of SEL may not be uniform but depend on student SEC levels: providing targeted SEL supports for students with lower SEC might improve their academic competence and peer relationships, while students with typical SEC can also benefit from SEL interventions by additionally fostering greater academic autonomy. However, for SEL to reach its full potential, focusing solely on individual SEC development would not be enough; broader systemic and societal changes are essential [85]. This includes shifting from a competitive to a more collaborative school environment and valuing diverse talents and holistic development. Future research should explore how school-based SEL can drive both overall improvements and disparity reduction in social–emotional outcomes, ensuring that all students have equitable opportunities to thrive.

### 4.3. Limitations and Recommendations for Future Research

One important limitation to note is the operationalization of the study variables. Due to the use of secondary data, the variables included in our model were measured using the relevant indicators among the existing data. Specifically, the indicators used to measure SEC, and the three mediators were not standardized assessments that have previously been tested and validated. This might have led to the results of generally small explanatory power for both depression and life satisfaction (both R^2^ = 0.09) in the path model. Although the model fit was good and basic psychometric properties (e.g., factor analysis results and Cronbach’s alpha) were examined to be acceptable, we call for future research that examines SEC growth profiles and the proposed path model using more established and validated measures. Besides, although depression and life satisfaction represent two core indicators of negative and positive mental health, respectively, we acknowledge that the use of secondary data limits the scope of our investigation, especially constraining our ability to examine other important indicators of adolescent mental health, such as other internalizing and externalizing behavioral problems. Relatedly, all of the variables were self-reported. Although it is aligned with SDT, which emphasizes the importance of subjective perceptions of autonomy, competence, and relatedness [41], the use of multi-informant and multi-method measurements would have strengthened the analysis.

It is also worthwhile to mention that the data used in the analysis were collected from 2018 to 2022, which includes the COVID-19 pandemic era. In line with previous studies conducted prior to the pandemic, our LPGM results showed generally consistent patterns of SEC development in the upper elementary school years. Further, no drastic differences in SEC growth trajectories were observed between waves 1 and 2 (2018 to 2019) and waves 2 and 3 (2019 to 2020, including the outbreak of COVID-19). Yet, it is important to acknowledge that the pandemic, as a global public health crisis, might have had some profound impacts on mediators, outcomes, and other confounding variables that are not fully captured in the current analysis. For example, existing research has suggested disruptions to regular schooling and online course-taking could have influenced students’ academic functioning, while inadequate physical activities and the elevated feelings of uncertainty, panic, and anxiety may also influence peer relationships and other mental health outcomes [86,87,88]. To better understand how pandemic-related changes in multi-systemic contexts (e.g., school, home, policy) influenced adolescents’ developmental trajectories and well-being outcomes, more research is needed to disentangle the pandemic effects from normative developmental changes—for example, by comparing these relationships before, during, and after the pandemic across different age cohorts.

Furthermore, even though we examined a theory-informed model with longitudinal data while statistically controlling for potential confounders and covariates, our findings do not guarantee a causal inference. Conducting a rigorous experimental study (e.g., a randomized controlled trial of an SEL intervention) might be useful to examine the long-term effects of promoting SEC on mental health outcomes and to investigate the underlying mediational pathways.

## 5. Conclusions

This study provides evidence on the effects of SEC growth profiles on adolescents’ mental health outcomes, using five years of nationally representative data on South Korean youth. The overall levels of self-management and group collaboration during the upper elementary school years were found to predict later depression and life satisfaction of Korean middle school students. Academic competence and peer relatedness were found to play a mediating role in explaining the effects of earlier SEC on later mental health outcomes during the middle school transition period. In addition, academic autonomy was another significant mediator, explaining the effects of having higher-than-average levels of SEC in elementary school years on better mental health outcomes later.

As mentioned earlier, Korean adolescents face growing concerns about mental health challenges [89,90,91,92], which is not just limited to one single country. We hope the findings of this study inform future research and practice efforts to prevent mental health problems and promote mental well-being among adolescents from Korea and other countries.

## Data Availability

The National Youth Policy Institute of South Korea provides publicly available de-identified datasets from this survey (National Youth Policy Institute, 2023); more information can be found in https://www.nypi.re.kr/archive/eps (accessed on 29 October 2025).

## Supplementary Materials

The following supporting information can be downloaded at: https://www.mdpi.com/article/10.3390/ijerph22111744/s1, Table S1: Sociodemographic Characteristics of Participants at Baseline; Table S2: Descriptive Statistics of Outcomes, Predictors, and Mediators; Table S3: Bivariate Correlations among the Study Variables; Table S4: Model Fit of Latent Profile Growth Modeling for SEC (*N* = 2607); Table S5: Descriptive Analysis and Comparison of Baseline Measures Across the Three Subgroups; Table S6: Regression Coefficients for Direct, Indirect, and Total Effects.

## Abbreviations

The following abbreviations are used in this manuscript:

AIC	Akaike information criterion
aBIC	adjusted Bayesian information criteria
BLRT	Bootstrap likelihood ratio test
BIC	Bayesian information criteria
CFI	Comparative fit index
CI	Confidence interval
FIML	Full information maximum likelihood
LMRT	Lo–Mendell–Rubin likelihood ratio test
LPGM	Latent profile growth modeling
MCAR	Missing completely at random
MLR	Maximum likelihood with robust standard errors
RMSEA	Root-mean-square error of approximation
SDT	Self-determination theory
SEC	Social–emotional competence
SEL	Social–emotional learning
SRMR	Standardized root-mean-square residual
TLI	Tucker–Lewis index
KCYPS	Korean Children and Youth Panel Survey
