# Supplementary material for: Social–Emotional Competence Growth Profiles in Upper Elementary School Years and Pathways to Mental Health Outcomes in Middle School
Source: Int J Environ Res Public Health. 2025 Nov 18;22(11):1744. doi: 10.3390/ijerph22111744 (PMC12652882; doi:10.3390/ijerph22111744)
Supplement: Supplementary file 1 [file ijerph-22-01744-s001.zip › ijerph-3927820-supplementary/Table S1.pdf]

Table S1

Sociodemographic Characteristics of Participants at Baseline

Variable	Range	*M*	*SD*	*n*	%
Age (in year)	[9, 11]	10	.10	—	—
Household size	[2, 8]	4.16	.88	—	—
Gender					
Girl	—	—	—	1294	49.64
Boy	—	—	—	1313	5.36
Missing	—	—	—	0	0.00
Physical health	[1, 4]	3.41	.60	—	—
Number of siblings	[1, 6]	2.12	.72	—	—
Family income level (unit 10,000 KRW)					
No income	—	—	—	6	0.23
Below 100	—	—	—	32	1.23
100-200	—	—	—	111	4.27
200-300	—	—	—	233	8.95
300-400	—	—	—	478	18.37
400-500	—	—	—	544	2.91
500-600	—	—	—	484	18.60
600-700	—	—	—	259	9.95
700-800	—	—	—	162	6.23
800-900	—	—	—	91	3.50
900-1000	—	—	—	77	2.96
Above 1000	—	—	—	125	4.80
Missing	—	—	—	5	0.20
Parent-perceived family socioeconomic level					
Very low	—	—	—	36	1.39
Low	—	—	—	305	11.75
Middle	—	—	—	2020	77.81
High	—	—	—	227	8.74
Very high	—	—	—	8	0.31
Missing	—	—	—	11	0.40
Region					
City				2195	84.20
Rural				412	15.80
Missing				0	0.00
Total				2607	10.00
