# Supplementary material for: Social–Emotional Competence Growth Profiles in Upper Elementary School Years and Pathways to Mental Health Outcomes in Middle School
Source: Int J Environ Res Public Health. 2025 Nov 18;22(11):1744. doi: 10.3390/ijerph22111744 (PMC12652882; doi:10.3390/ijerph22111744)
Supplement: Supplementary file 1 [file ijerph-22-01744-s001.zip › ijerph-3927820-supplementary/Table S2.pdf]

Table S2

Descriptive Statistics of Outcomes, Predictors, and Mediators

Variable	Cronbach's α	*Mean*	*SD*	Skewness	Kurtosis	Missing *(%)*
Outcomes (Wave 5, Grade 2 in middle school)						
Depression (10 items)	.89	1.75	.53	.64	.39	11.35
Life satisfaction (5 items)	.81	2.68	.53	-.07	.49	11.35
Predictors (Wave 1, Grade 4 in elementary school)						
Self-management (21 items)	.89	3.03	.46	-.09	-.35	0.00
Group collaboration (14 items)	.93	3.03	.54	-.29	.40	0.00
———— (Wave 2, Grade 5 in elementary school)						
Self-management (21 items)	.89	2.93	.44	-.03	-.32	6.52
Group collaboration (14 items)	.93	2.97	.51	-.35	.75	6.52
———— (Wave 3, Grade 6 in elementary school)						
Self-management (21 items)	.89	2.83	.46	.06	-.52	7.52
Group collaboration (14 items)	.92	2.96	.47	-.07	.73	7.52
Mediators (Wave 4, Grade 1 in middle school)						
Academic autonomy (4 items)	.80	3.08	.58	-.27	-.14	12.74
Academic competence (2 items)	.78	3.57	.72	-.28	.38	17.49
Peer relatedness (8 items)	.87	3.01	.50	-.33	.86	12.74
