# Supplementary material for: Social–Emotional Competence Growth Profiles in Upper Elementary School Years and Pathways to Mental Health Outcomes in Middle School
Source: Int J Environ Res Public Health. 2025 Nov 18;22(11):1744. doi: 10.3390/ijerph22111744 (PMC12652882; doi:10.3390/ijerph22111744)
Supplement: Supplementary file 1 [file ijerph-22-01744-s001.zip › ijerph-3927820-supplementary/Table S3.pdf]

Table S3

Bivariate Correlations among the Study Variables

Variable	1	2	3	4	5	6	7	8	9	10	11	12	13	14	15
Wave 1 (Grade 4 in elementary school)															
1 Self-management	—														
2 Group collaboration	.52***	—													
3 Depression	-.59***	-.39***	—												
4 Life satisfaction	.47***	.45***	-.48***	—											
5 Academic competence	.34***	.31***	-.25***	.34***	—										
6 Peer relatedness	.30***	.53***	-.27***	.34***	.19***	—									
Wave 2 (Grade 5 in elementary school)															
7 Self-management	.44***	.28***	-.29***	.27***	.20***	.15***	—								
8 Group collaboration	.23***	.35***	-.16***	.20***	.16***	.23***	.43***	—							
Wave 3 (Grade 6 in elementary school)															
9 Self-management	.35**	.20***	-.21**	.21***	.13***	.13***	.46**	.21***	—						
10 Group collaboration	.23**	.34***	-.16**	.19***	.16***	.23***	.25***	.40***	.36***	—					
Wave 4 (Grade 1 in middle school)															
11 Academic autonomy	.20***	.18***	-.14***	.14***	.16***	.11***	.18***	.15***	.24***	.19***	—				
12 Academic competence	.16***	.18***	-.11***	.14***	.26***	.10***	.16***	.14***	.15***	.18***	.29***	—			
13 Peer relatedness	.12***	.20***	-.11***	.12***	.05*	.25***	.11***	.19***	.12***	.25***	.26***	.16***	—		
Wave 5 (Grade 2 in middle school)															
14 Depression	-.13***	-.11***	.18***	-.13***	-.07***	-.09***	-.17***	-.09***	-.23***	-.13***	-.13***	-.14***	-.11***	—	
15 Life satisfaction	.12***	.11***	-.12***	.14***	.07**	.06**	.20***	.13***	.19***	.16***	.12***	.23***	.13***	-.35***	—

Note. * $p < .05$, ** $p < .01$, *** $p < .001$ (two-tailed)
