# Supplementary material for: Social–Emotional Competence Growth Profiles in Upper Elementary School Years and Pathways to Mental Health Outcomes in Middle School
Source: Int J Environ Res Public Health. 2025 Nov 18;22(11):1744. doi: 10.3390/ijerph22111744 (PMC12652882; doi:10.3390/ijerph22111744)
Supplement: Supplementary file 1 [file ijerph-22-01744-s001.zip › ijerph-3927820-supplementary/Table S4 [revised].pdf]

Table S4

Model Fit of Latent Profile Growth Modeling for SEC (N = 2,607)

	AIC	BIC	aBIC	LMRT	BLRT	Entropy	Group size n *(%)*					
							1	2	3	4	5	6
1-profile	20302.614	20361.274	20329.501	—	—	—	2607 (100)					
2-profile	18186.819	18274.808	18227.149	0.000	0.000	0.693	1582 (61)	1025 (39)				
3-profile	*17773.486*	*17890.805*	*17827.259*	*0.0004*	*0.000*	*0.648*	*1446 (55)*	*682 (27)*	*479 (18)*			
4-profile	17566.393	17713.042	17633.609	0.0001	0.000	0.692	332 (13)	1067 (41)	70 (3)	1138 (44)		
5-profile	17415.305	17591.283	17495.965	0.0006	0.000	0.655	735 (28)	412 (16)	67 (3)	1007 (39)	386 (15)	
6-profile	17321.203	17526.512	17415.307	0.0028	0.000	0.708	719 (28)	63 (2)	29 (1)	360 (14)	409 (16)	1027 (39)

Note. In the 3-profile solution, the original group sizes were 55.47%, 27.08%, and 17.45%, respectively. For ease of interpretation, the values are rounded to whole numbers.
