# Supplementary material for: Social–Emotional Competence Growth Profiles in Upper Elementary School Years and Pathways to Mental Health Outcomes in Middle School
Source: Int J Environ Res Public Health. 2025 Nov 18;22(11):1744. doi: 10.3390/ijerph22111744 (PMC12652882; doi:10.3390/ijerph22111744)
Supplement: Supplementary file 1 [file ijerph-22-01744-s001.zip › ijerph-3927820-supplementary/Table S5 [revised].pdf]

Table S5

Descriptive Analysis and Comparison of Baseline Measures Across the Three Subgroups

Variable	Subgroup						F-statistic	p-value
	High SEC		Medium SEC		Low SEC			
	M	*SD*	*M*	*SD*	*M*	*SD*		
AC1	4.38	0.91	4.02	1.11	3.67	1.30	132.7	<.001***
PR1	3.27	0.48	3.02	0.48	2.72	0.53	18.2	<.001***
Dp1	1.20	0.32	1.45	0.46	1.91	0.57	351.9	<.001***
LS1	3.47	0.45	3.15	0.51	2.76	0.57	271.7	<.001***
Gender	0.57	0.50	0.50	0.50	0.45	0.50	7.95	<.001***
Health	3.64	0.50	3.44	0.57	3.44	0.57	89.19	<.001***
Siblings	2.09	0.72	2.11	0.72	2.16	0.74	1.82	0.162
Income	7.07	2.29	6.62	2.23	6.25	2.22	19.09	<.001***
Region	0.86	0.34	0.85	0.36	0.82	0.39	2.88	0.057
Parenting	3.72	0.31	3.50	0.48	3.22	0.49	213.9	<.001***
Teacher	3.32	0.43	3.01	0.44	2.73	0.47	245.3	<.001***
SM1	3.49	0.31	3.08	0.35	2.56	0.31	1133	<.001***
SM2	3.43	0.81	2.93	0.81	2.54	0.69	1010	<.001***
SM3	3.30	0.95	2.81	0.79	2.51	0.78	582.4	<.001***
GC1	3.51	0.41	3.06	0.43	2.52	0.42	777.8	<.001***
GC2	3.43	0.87	2.96	0.48	2.59	0.78	483.9	<.001***
GC3	3.38	0.99	2.94	0.83	2.65	0.85	403.9	<.001***

Note. AC1 = academic competence at baseline; PR1 = peer relatedness at baseline; Dp1 = depression at baseline; LS1 = life satisfaction at baseline; Gender: using boys as the reference group (girl = 1, boy = 0); Health = physical health; Siblings = number of siblings in Wave 2 (we used this measure as the variable was not asked at baseline); Income = parent-reported family income level; Region: using rural as the reference group (city = 1, rural = 0); Parenting = student-reported positive parenting; Teacher = teacher relationships; SM1-SM3 = self-management in Wave 1 to 3; GC1-GC3 = group collaboration in Wave 1 to 3.
