# Supplementary material for: Social–Emotional Competence Growth Profiles in Upper Elementary School Years and Pathways to Mental Health Outcomes in Middle School
Source: Int J Environ Res Public Health. 2025 Nov 18;22(11):1744. doi: 10.3390/ijerph22111744 (PMC12652882; doi:10.3390/ijerph22111744)
Supplement: Supplementary file 1 [file ijerph-22-01744-s001.zip › ijerph-3927820-supplementary/Table S6 [revised].pdf]

Table S6

Regression Coefficients for Direct, Indirect, and Total Effects

Path	Coefficient Estimate					Bootstrapping (*95%* CI)	
	Std.	*Un.std*	*SE*	*Z*	*p-value*	*Lower*	*Upper*
Direct effects of predictors on outcomes							
High→Dp5	-.121	-.164	.030	-5.443	.000	-.222	-.103
Low→Dp5	.55	.067	.032	2.129	.033	.008	.128
High→LS5	.084	.114	.033	3.422	.001	.051	.177
Low→LS5	-.073	-.090	.030	-2.986	.003	-.150	-.035
Direct effects of predictors on mediators							
High→AA4	.114	.166	.034	4.813	.000	.100	.234
Low→AA4	-.123	-.163	.032	-5.164	.000	-.225	-.101
High→AC4	.100	.182	.045	4.067	.000	.087	.263
Low→AC4	-.050	-.083	.039	-2.132	.033	-.160	-.005
High→PR4	.093	.116	.031	3.705	.000	.055	.177
Low→PR4	-.069	-.079	.027	-2.896	.004	-.132	-.028
Direct effects of mediators on outcomes							
AA4→Dp5	-.046	-.043	.022	-1.953	.051	-.089	-.002
AC4→Dp5	-.077	-.057	.019	-3.086	.002	-.094	-.020
PR4→Dp5	-.067	-.072	.025	-2.882	.004	-.121	-.022
AA4→LS5	.020	.019	.023	.828	.408	-.026	.064
AC4→LS5	.180	.134	.018	7.634	.000	.098	.166
PR4→LS5	.064	.069	.027	2.582	.010	.016	.119
Indirect effects of main interest							
High→AA4→Dp5	-.005	-.007	.004	-1.774	.076	-.016	.000
High→AC4→Dp5	-.008	-.01	.004	-2.397	.017	-.020	-.003
High→PR4→Dp5	-.006	-.008	.004	-2.314	.021	-.016	-.002
Low→AA4→Dp5	-.002	-.003	.004	-.823	.411	-.011	.004
Low→AC4→Dp5	-.009	-.011	.005	-2.047	.041	-.023	-.001
Low→PR4→Dp5	-.004	-.005	.003	-1.738	.082	-.013	-.001
High→AA4→LS5	.006	.007	.004	1.775	.076	.000	.016
High→AC4→LS5	.004	.005	.003	1.794	.073	.000	.011
High→PR4→LS5	.005	.006	.003	1.96	.050	.001	.012
Low→AA4→LS5	.002	.003	.004	.812	.417	-.004	.011
Low→AC4→LS5	.018	.024	.007	3.576	.000	.011	.038
Low→PR4→LS5	.006	.008	.004	2.114	.035	.001	.016
Total effects of main interest							
High→Dp5	-.141	-.190	.030	-6.26	.000	-.249	-.130
High→LS5	.110	.150	.035	4.288	.000	.083	.217
Low→Dp5	.069	.085	.031	2.697	.007	.027	.144
Low→LS5	-.089	-.110	.030	-3.673	.000	-.170	-.055
Direct effects of covariates on mediators and outcomes							
Dp1→Dp5	.098	.098	.026	3.783	.000	.043	.149
Gen→Dp5	.080	.086	.025	3.518	.000	.038	.134
PH1→Dp5	-.053	-.047	.022	-2.153	.031	-.092	-.003

Sibg2→Dp5	.000	.000	.016	0.016	.987	-.034	.030
Inc1→Dp5	.019	.005	.005	0.875	.382	-.006	.014
City1→Dp5	.036	.053	.032	1.628	.103	-.009	.116
SPP1→Dp5	-.006	-.007	.03	-0.238	.812	-.067	.057
TR1→Dp5	.021	.023	.029	0.810	.418	-.032	.080
LS1→LS5	.036	.034	.025	1.390	.164	-.016	.083
Gen→LS5	-.062	-.067	.023	-2.905	.004	-.114	-.022
PH1→LS5	.001	.001	.02	0.057	.954	-.040	.041
Sibg2→LS5	.011	.008	.017	0.490	.624	-.025	.042
Inc1→LS5	.002	.001	.005	0.097	.922	-.010	.011
City→LS5	-.026	-.038	.031	-1.224	.221	-.101	.022
SPP1→Ls5	.038	.047	.031	1.499	.134	-.017	.107
TR1→LS5	-.011	-.013	.028	-0.443	.658	-.069	.044
Gen→AA4	.044	.051	.025	2.024	.043	.004	.103
PH1→AA4	.025	.024	.021	1.132	.258	-.017	.065
Sibg2→AA4	-.034	-.027	.018	-1.509	.131	-.063	.008
Inc1→AA4	.117	.030	.006	5.385	.000	.019	.041
City→AA4	-.038	-.061	.033	-1.808	.071	-.125	.007
SPP1→AA4	.043	.056	.032	1.761	.078	-.010	.120
TR1→AA4	.018	.021	.03	0.715	.474	-.036	.083
AC1→AC4	.178	.171	.021	8.038	.000	.131	.214
Gen→AC4	-.020	-.029	.030	-0.943	.346	-.086	.033
PH1→AC4	.031	.037	.028	1.330	.184	-.017	.09
Sibg2→AC4	-.024	-.024	.022	-1.106	.269	-.066	.019
Inc1→AC4	.072	.023	.007	3.128	.002	.009	.039
City→AC4	-.008	-.016	.039	-0.420	.674	-.093	.064
SPP1→AC4	-.008	-.014	.044	-0.314	.754	-.098	.078
TR1→AC4	.064	.096	.038	2.508	.012	.020	.174
PR1→PR4	.203	.194	.024	8.176	.000	.152	.240
Gen→PR4	.090	.090	.021	4.237	.000	.049	.134
PH1→PR4	.004	.003	.018	0.168	.867	-.035	.039
Sibg2→PR4	.000	.000	.014	0.010	.992	-.029	.027
Inc1→PR4	.018	.004	.005	0.837	.402	-.005	.014
City→PR4	-.012	-.016	.027	-0.598	.550	-.071	.038
SPP1→PR4	-.005	-.006	.028	-.215	.830	-.059	.049
TR1→PR4	.001	.002	.027	0.057	.954	-.052	.054

Note. Total *N* = 2,607. CI = confidence interval; Lower = lower limit; Upper = upper limit. Abbreviations: Dp5 = depression in Wave 5; LS5 = life satisfaction in Wave 5; High = Profile with high SEC (in reference to Profile with medium SEC); Low = Profile with low SEC (in reference to Profile with medium SEC); AA4 = academic autonomy in Wave 4; AC4 = academic competence in Wave 4; PR4 = peer relatedness in Wave 4; AC1 = academic competence at baseline; PR1 = peer relatedness at baseline; Dp1 = depression at baseline; LS1 = life satisfaction at baseline; Gen = gender (girl = 1, boy = 0); Inc1 = parent-reported family income level in Wave1; SPP1 = student-reported positive parenting in Wave 1.
